# Improved beekeeping practices, honey bee flora potential and flowering calendar in South Ethiopia

**DOI:** 10.1371/journal.pone.0304259

**Published:** 2024-05-29

**Authors:** Markos Fisaha Delena, Asrat Diriba Asfaw

**Affiliations:** 1 School of Animal and Range Sciences, College of Agriculture, Hawassa University, Hawassa, Ethiopia; 2 Department of Animal Sciences, College of Agriculture and Natural Resource, Dilla University, Dilla, Ethiopia; Khwaja Fareed University of Engineering & Information Technology, PAKISTAN

## Abstract

In Ethiopia, improved hive technology dissemination was started before five-decades. However, the adoption of improved beekeeping technology is still very low. This study was conducted with the main objectives to evaluating improved beekeeping adoption level and honey yields of different hives and identification of major honey bee plants and flora calendar in the Gedeo zone, South Ethiopia. Three districts were selected purposively based on beekeeping potential and the number of improved hives own by beekeepers. The data was collected from 180 respondents using cross-sectional survey. The data was analyzed by using descriptive statistics such as mean, frequency and percentage and ANOVA. The result shown that the compositions of disseminated hives in the entire sampled respondents were 286, 476, 121 and 1494 Zander hive, Kenyan top bar hive (KTBH), Mud/Ethio-Ribrab hive (ERH) and Traditional hives respectively. Traditional beekeeping was the dominant system with 63% and intermediate followed by 25%, while modern beekeeping was only 12%. Based on overall mean honey yield, there was no significant difference (P = 0.244) between Zander and KTBH. However, the average honey yield of these improved hives were significantly (P<0.05) higher than Mud/ERH and Traditional hives. Gedeo zone had rich floral resource and diverse floral calendar. *Hygenia abyssinica*, *Bidens ghedoensis*, *Erythrinia abyssinica*, *Eucalyptus species*, *Cordia africana*, *Coffee arabica*, *Vernonia species*, *Susbania susban* and *Persea americana* were major honey bee flora in Gedeo zone. February-March was major honey harvesting season while May-July and October-December respectively were minor honey harvesting periods. Nevertheless, the majority of beekeepers have been practicing honey harvesting once a year from all hives due to lack of awareness and practical skills. Therefore, we recommend that the local government should focus on educating beekeepers to enable them utilizing exhaustively the opportunities of multi-floral season and improved hive technology to maximize honey yield in the area.

## 1 Introduction

**Fig 1:**
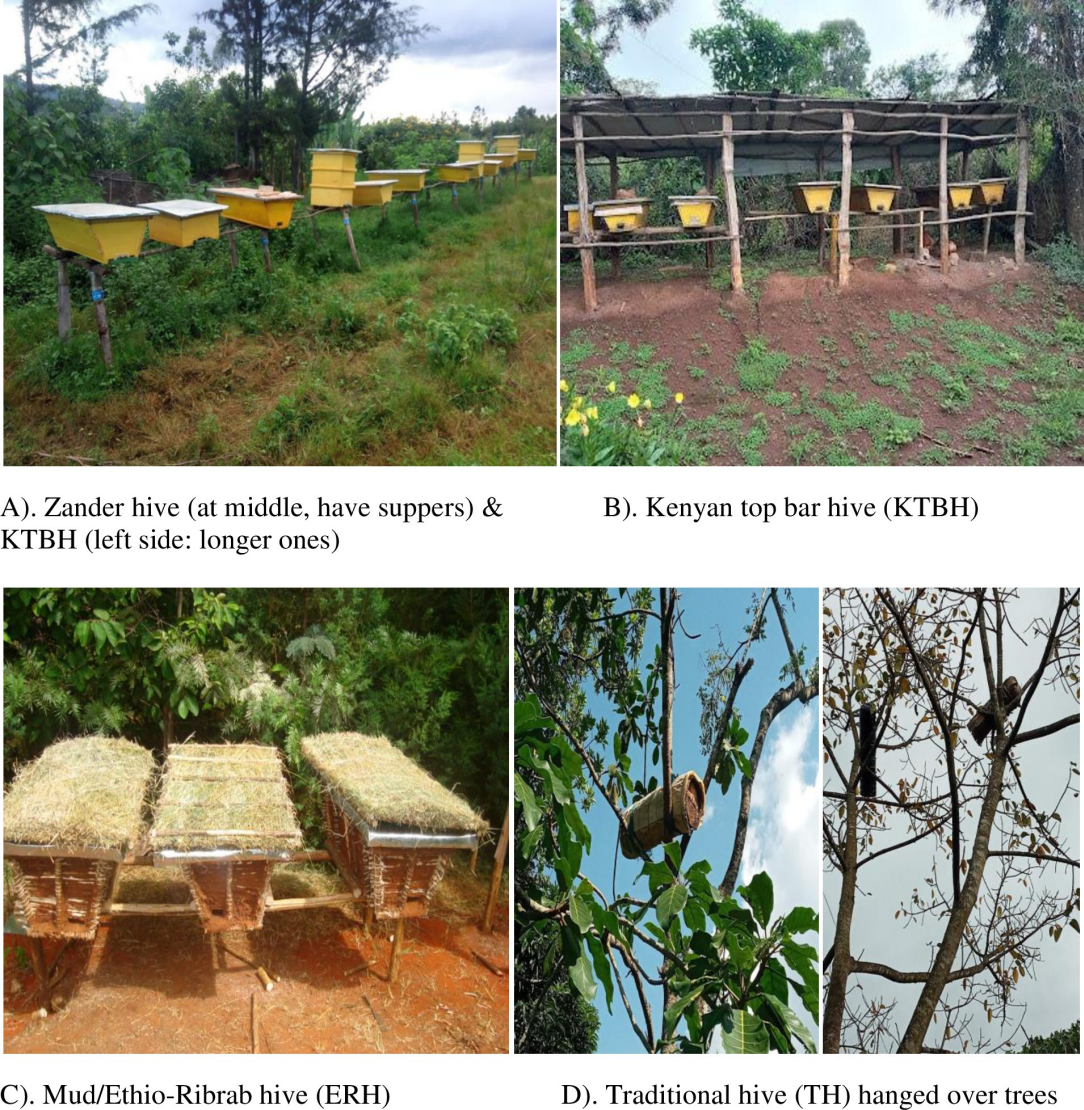
Types of hive in Gedeo Zone.

Beekeeping is an ancient agriculture practiced for more than five thousand years in the basket hives in Ethiopia [1–3]. Even if beekeeping is a long-standing business [4], the sector remains subsistent traditional and underdeveloped [5] due to low level of technology adoption [6] and the absence of honeybee breeding and improvement programs [7, 8]. In Ethiopia, beekeeping is commonly practiced in diverse types of hives: traditional, transitional (intermediate), and modern [9]. Traditional beekeeping is practiced throughout all regions of the country in a wide range of hives made from locally available materials [10]. Traditional hives are cylindrical in shape and small in size, which makes it difficult to carry out internal hive inspection because the combs are fixed to the hive body and not suitable to use a queen excluder to separate honey from broods. As a result, traditional hives (Fig 1D) are commonly characterized by low honey yield and poor quality [11].

Modern and transitional hive technologies were introduced to Ethiopia since 1970 and 1976 respectively [12, 13]. Despite the long history of improved hive technology dissemination to farmers over the last five decades, the share of improved hives remains low [14]. According to a recent report by the Ethiopian Federal Democratic Republic Central Statistics Agency [15], 96% of colonies were managed in the traditional system, while only 2.8% were in the modern hives, and the remaining 1.1% were kept in the intermediate hives. In general, a lack of skilled manpower, the absence of research and training institutions supporting the beekeeping sub-sector, poor extension service, and the high cost of beekeeping equipment are major factors in the lower level of technology adoption [16].

Transitional beekeeping system is a system in between traditional and box hives or modern system [12]. There are different types of transitional beehives practiced widely in Ethiopia such as Kenya top-bar hives (KTBH) (Fig 1B), Tanzania top-bar hive (TTBH), Mud-block hives and Ethio-Ribrab hive (ERH) (Fig 1C). Among transitional bee hives KTBH is widely adopted due to its low cost and easy for construction [12]. KTBH hive carries specially designed 27–30 pieces of timber top bars where honeybees attach their combs. The bars have 3.2 cm width and 48.3 cm length [17]. The top bars are easily removable and that makes beekeepers to work fast during hive inspection and honey harvesting [13]. The average honey yield of transitional (KTBH) is between 15 to 20kg/hive per year [6]. Unlike to KTBH, Ethio-Ribrab hive constructed by any locally available materials in the same design with KTBH and give the same honey yield per harvest with KTBH. The advantage of ERH over KTBH is the construction cost is very cheap.

The modern beekeeping system consists of a rectangular box-hives overlaid one above the other [12]. The population of bees and the seasons determines the number of box-hives superimposed. Modern hives such as Zander (Fig 1A) and Langstroth were introduced to Ethiopia in 1970 and distributed widely since then [12, 13]. The box hive beekeeping system is modern techniques [18] and this hive yields on average 30 kg/year per hive [6]. Modern beekeeping system has many advantages over traditional and transitional beekeeping system. It gives higher honey yield and better quality, it allows all range of management to maximize the productivity of colony such as regular inspection, volume adding and reducing and swarm controlling and it is suitable to move the colony from place to place for pollination service. However, box hive (frame hive) are relatively requires the higher investment cost and trained manpower to run beekeeping business with it [12, 13].

Ethiopia is endowed with over 1500 species of honey bee plants, which result in a wide range of agro-ecological diversity [19, 20]. The honey bee plants are sources of forage for honeybees as nectar, pollen, or both [1, 19]. According to [21], all bee plants are not equally important to bees and honey production. However, some supply both nectar and pollen abundantly, while others provide only nectar or pollen [22]. Depending on farming practices and the agro-climate in which they grow, bee plants and their blooming periods differ from place to place [23]. Thus, a deep understanding of floral resources, their quality, and the flowering season helps beekeepers to utilize the potential of the areas to the highest level and hence increase beekeeping productivity [19].

Gedeo Zone has a great diversity of flowering plant species, which comprises forest trees, agro-forestry, horticultural fruits, and shrubs that are potentially useful for beekeeping. Despite the potential of the area, beekeeping productivity is low due to low technological application and a lack of practical skills in improved beekeeping. Improved beekeeping technology was newly introduced in the Gedeo zone. There have been very limited scientific studies conducted on improved beekeeping adoption status and the identification and documentation of honeybee flora in the area. Most flora identifications in Ethiopia are limited to the central and western parts of the country [20]. Therefore, this study was mainly focused on evaluating the improved beekeeping adoption level and honey yields of different hives and the identification of major honey bee plants and their flowering calendar in the Gedeo zone.

## 2. Materials and methods

### 2.1. Description of study area

The Gedeo Zone is located in southern Ethiopia, 365 kilometers (km) from Addis Ababa and 90 kilometers from Hawassa, the capital of Sidama Region. The altitude of the area ranges from 1,268 to 2,993 meters above sea level [24]. The zone has an average temperature of 21.5°C and receives 1500mm of mean annual rainfall. The rainfall pattern is bimodal, with a short period (30%) between March and May and a long period of rainfall (60%) between July and October. The agro-climate of the zone consists of 67% mid-altitude, 30% Highland, and 3% lowland [25, 26].

### 2.2. Sample size and sampling technique

To meet the objective of this study, three districts, namely Kochore, Wonago, and Bule, were selected purposefully out of seven districts in the Gedeo zone based on the beekeeping potential and the number of improved hives owned by beekeepers. Three kebeles (the smallest administration unit under the district), which had large beekeeping potential and improved hives widely disseminated were purposefully selected from each district. The total number of 20 beekeepers who own improved hives (frame hives or Kenyan top bar hives) was purposefully selected from each Kebele to know the adoption level of improved hive technology, and the total sample size included in this study was 180 respondents.

### 2.3. Data collection method

Before conducting the survey, discussions were held with district beekeeping experts, extension workers, and knowledgeable local leaders to select potential Kebeles and respondents. The survey questionnaire was used to collect the primary data from the beekeepers. The pilot survey was conducted to pre-test the survey questionnaire before the actual survey, and the actual survey was conducted after reviewing the questionnaire based on the results of the pilot survey.

### 2.4. Data analysis

The data generated through the survey questionnaire was analyzed using SPSS software version 20 package and Microsoft Excel. Descriptive statistics such as mean, percentages, frequencies, figures and tables were used to describe respondents’ socio-economic characteristics. One way ANOVA were used to test mean variance and the significance of mean separated by LSD of mean at 0.05 level of significance.

### 2.5. Ethical clearance statement

To conduct the present study entitled “Improved beekeeping practices, honey bee flora potential and flowering calendar in South Ethiopia” permission (the need for consent was waived) was received from institutional review board of Dilla University (DU/4-8/1303). In addition, the oral informed consent was received from all participants before data collection. The objectives of the study were explained to all participants, and all had the right to withdraw from the research if they not feel comfortable. They were assured that the principle of confidentiality would be respected and this study only used for academic publication.

## 3. Result

### 3.1 Characteristics of respondents

As shown in the result, beekeepers had low experience in both traditional and improved beekeeping. The majority (77.8%) of respondents who practiced traditional beekeeping and all respondents who practiced improved beekeeping (in Zander and Kenyan top bar hives) had less than ten years experience (Table 1). The average colony size per individual apiary site was 6.4 and the mean age of respondents was 41 years. The majority of respondents (68%) attended primary school. The result further indicated that, male was dominating beekeeping in the study area.

**Table 1:** Respondents’ characteristics in the study area.

Beekeeping experience (years)	<5	5–10	10–15	>15
Experience in traditional BK%(n)	40.6% (73)	37.2% (67)	10% (18)	12.2% (22)
Experience in improved BK%(n)	78.3% (141)	21.6%(39)	-	-
Colony size per individual (mean)	6.4			
Mean Age (year)	40.9			
Education (%)				
Illiterate	9.4			
1–4	20.0			
5–8	48.3			
9–12	20.6			
Certificate and above	1.7			
Sex (%)				
Male	86.7			
Female	13.3			

*BK = beekeeping*, *n = number of respondents*

### 3.2 Beekeeping proportion and average honey yield in different hives

The survey result shows that the total number of beehives in the entire sample of respondents was 2377. Of which 1494, 476, 286 and 121 were traditional, KTBH, Zander and Mud/Ethio-Ribrab hives respectively. Out of total colonized hives, 150 (52.6%) of Zander, 283 (59.5%) of KTBH, and 728 (48.7%) of traditional hives were occupied with colonies. In general, only about fifty percent of overall hives were occupied with colonies, while the remaining 50% were empty due to various reasons. On the other hand, Kochore district was superior with a proportion of colony-occupied hives of 67.5 and 62.1 percent in the Zander and KTB hives respectively compared with 43.5 and 64.2 percent respectively in Bule and 29.8 and 51.3 percent respectively in Wonago district (Table 2).

**Table 2:** Composition of hives and beekeeping proportion in sampled districts.

District	Type of hive	Total hive no.	CR	%	CL	%	Beekeeping
							Proportion
							(%)
Bule	Zander	39	17	43.5	22	56.5	9.2
	KTBH	190	122	64.2	68	35.7	44.9
	Mud /ERH	40	16	40	24	60	9.5
	TH	154	141	91.5	13	8.4	36.4
	Sub Total	423	296	69.9	127	30.0	100
Kochore	Zander	160	108	67.5	52	32.5	18.9
	KTBH	132	82	62.1	50	37.9	15.6
	Mud/ERH	15	0	0	15	100	1.8
	TH	538	312	57.9	226	42.0	63.7
	Sub Total	845	502	59.4	343	40.6	100
Wonago	Zander	87	26	29.8	61	70.2	7.8
	KTBH	154	79	51.3	75	48.7	13.9
	Mud/ERH	66	10	15.2	56	84.8	6
	TH	802	275	34.2	527	65.8	72.3
	Sub Total	1109	390	35.2	719	64.8	100
Grand total	Zander	286	150	52.6	108	47.4	12
	KTBH	476	283	59.5	193	40.5	20
	Mud/ERH	121	26	21.5	95	78.5	5
	TH	1494	728	48.7	766	51.3	62.9
	Total	2377	1187	49.9	1162	48.9	100

*KTBH = Kenyan top bar hive*, *ERH = Ethio-Ribrab hive and TH = Traditional hive CR = colony right hives*, *CL = colony less hives*

Regarding beekeeping proportion, traditional beekeeping was dominant with 63%, transitional beekeeping (KTBH and Mud/ERH) was followed by 25%, while colony managed in the Zander hive (modern hive) was only about 12% (Fig 1A). This result further revealed that the KTBH was more disseminated and colonized compared with the Zander hive (Table 2). It is clearly revealed that beekeepers have relatively better managed colonies in the KTBH than in the frame hive.

The ANOVA analysis result for honey yield shown the significant differences (P<0.005) among different hives in each sampled districts (Table 3). However, according to post hoc ANOVA analysis, there was no significant variation in honey yield between Zander and KTBH hives in all districts. The overall mean honey yield was highest (13.4 kg/hive/year) in the Zander hive, this followed by KTHB (12.5 kg/hive/year) and the lowest honey yield (5.5 kg/hive/year) was observed in the traditional hive (Table 3). Similar to the results observed in each district there was no significant difference (P = 0.244) on overall mean honey yield between Zander and KTBH. However, the average honey yields of these improved hives were significantly (P<0.05) higher than those of Mud/ERH and traditional hives (Table 3).

**Table 3:** Average honey yield (kg/hive/year) in different hives.

Districts	Hive type	Honey yield ($\bar{X}$)	Std. Deviation	Std. Error	F _value_	Sig.
Bule	Zander	7.5^a^	3.53	2.50	11.04	.000
	KTBH	11.1^a^	2.97	0.86		
	ETRH	12.8^b^	7.01	3.13		
	TH	6^ac^	2.26	0.42		
	Total mean	8.1	4.14	0.60		
Kochore	Zander	14.3^a^	4.35	0.73	94.78	.000
	KTBH	12.9^a^	3.06	0.85		
	ETRH	0	0	0		
	TH	5.5^b^	1.41	0.20		
	Total mean	9.7	5.17	0.52		
Wonago	Zander	13.3^a^	6.15	1.28	16.74	.000
	KTBH	12.9^a^	5.80	1.21		
	ETRH	5.6^b^	3.10	1.26		
	TH	5.1^b^	3.41	0.48		
	Total mean	9.0	6.15	0.63		
Over all mean	Zander	13.43^a^	5.04	0.76	67.66	.000
	KTBH	12.51^a^	4.56	0.65		
	Mud/ERH	8.86^b^	6.22	1.87		
	TH	5.5^c^	2.35	0.21		

*Different superscripts are significantly different at P<0.05* *(LSD)*

### 3.3 Major honey bee flora in the sampled areas

Among the 27 major honeybee flora species identified in the sampled area, 81.5%, 11%, and 7.5% were trees, shrubs, and weeds, respectively. According to the responses of the interviewed beekeepers, *Hygenia abyssinica*, *Bidens ghedoensis*, *Erythrinia abyssinica*, and *Eucalyptus* species, respectively were major honey plants in Bule district (Table 4). Likewise, *Cordia africana*, *Coffee arabica*, *Bidens ghedoensis*, *Vernonia* species, *Persea americana*, and *Susbania susban*, respectively, were dominant honey plants in the Kochore district. Whereas, *Coffee arabica* and *Cordia africana* were the dominant honey plants in Wonago district (Table 4). *Coffee arabica*, *Bidens ghedoensis*, *Cordia africana*, *Hygenia abyssinica*, *Eucalyptus* species, and apples were the common honeybee flora mentioned in all sampled districts with different levels of dominance.

**Table 4:** Major honeybee flora and flowering period as rated by beekeepers.

District	Scientific name	Common name	n*	Rank	Flowering period
Bule	*Hygenia abyssinica*	Koso	58	1	Oct-Feb
	*Erythrinia abyssinica*	Korch	38	3	Sept-Dec
	*Eucalyptus sp*.	Bahire zaf	35	4	March-April
	*Cytisus proliferus*	Tree lucern	22	5	Year round
	*Phytolacca dodecandra*	Endod	20	6	July-Oct
	*Aunona maricuta*	Gishta	15	7	Feb-April
	*Coffee Arabica*	Coffee	14	8	April-May
	*Apple*	Pome	12	9	Oct-Dec
	*Bidens ghedoensis*	Adey abeba	53	2	Sept-Dec
Kochore	*Cordia africana*	Wanza	52	1	Jan-July
	*Coffee Arabica*	Coffee	49	2	April-May
	*Croton macrostachys*	Bisana	29	7	April-July
	*Milletia ferruginea*	Birbra	24	8	Nov-April
	*Susbania susban*	Susbania	33	6	Dec-May
	*Vernonia sp*	Grawa	41	4	Dec-May
	*Syzygivm guineese*	Dokima	13	10	Dec-May
	*Apple*	Pome	6	11	Oct-Dec
	*Acacia Sp*.	Grar	3	12	March-Sept
	*Eucalyptus sp*.	Bahire zaf	19	9	March-April
	*Persea Americana*	Avocado	39	5	Sept-Dec
	*Bidens prestinaria*	Adey abeba	46	3	Sept -Oct
Wonago	*Coffee Arabica*	Coffee	47	1	April-May
	*Cordia africana*	Wanza	33	2	Jan-July
	*Croton macrostachys*	Bisana	4		April-July
	*Aunona maricuta*	Gishta	25	3	Feb-April
	*Hygenia abyssinica*	Koso	13	5	Sept-Oct
	*Apple*	Pome	4	8	Oct-Dec
	*Acacia Sp*.	Grar	5	7	March-Sept
	*Eucalyptus sp*.	Bahire zafi	11	6	March-April
	*Vernonia sp*	Raje	24	4	Dec-Feb

n* *= Number of beekeepers had each flora in their apiary site in each district*

### 3.4 Flora calendar in study area

The majority (59.4%) of the entire respondents reported that February–March was the main honey-harvesting period, and May–July and October–December respectively were minor honey harvesting seasons in the study area. However, the result further revealed that major honey harvesting seasons were different for mid-altitude and highland areas (Table 5).

**Table 5:** Beekeepers response on honey harvesting seasons in Gedeo Zone.

Harvesting Seasons						
Districts	Oct-Dec		Feb-March		May-July	
	n	%	n	%	n	%
Bule	33	55	1	1.7	26	43.3
Kochore	-	-	58	96.7	2	3.3
Wonago	2	3.3	48	80	10	16.7
Total (N = 180)	35	19.4	107	59.4	38	21.1

n *= Number of Beekeepers harvested honey*

**Fig 2:**
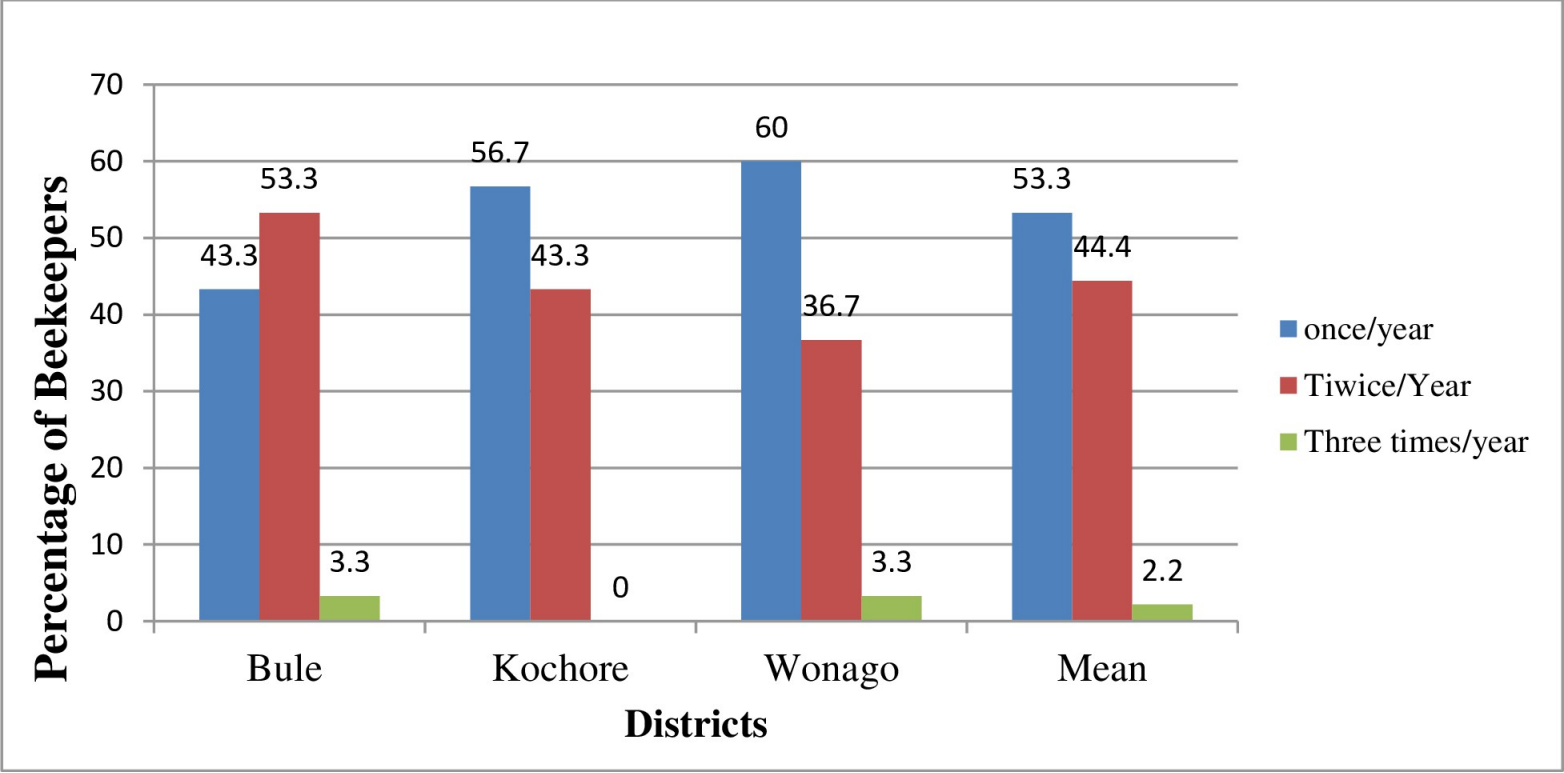
Beekeepers response on honey harvesting frequency (%).

**Fig 3:**
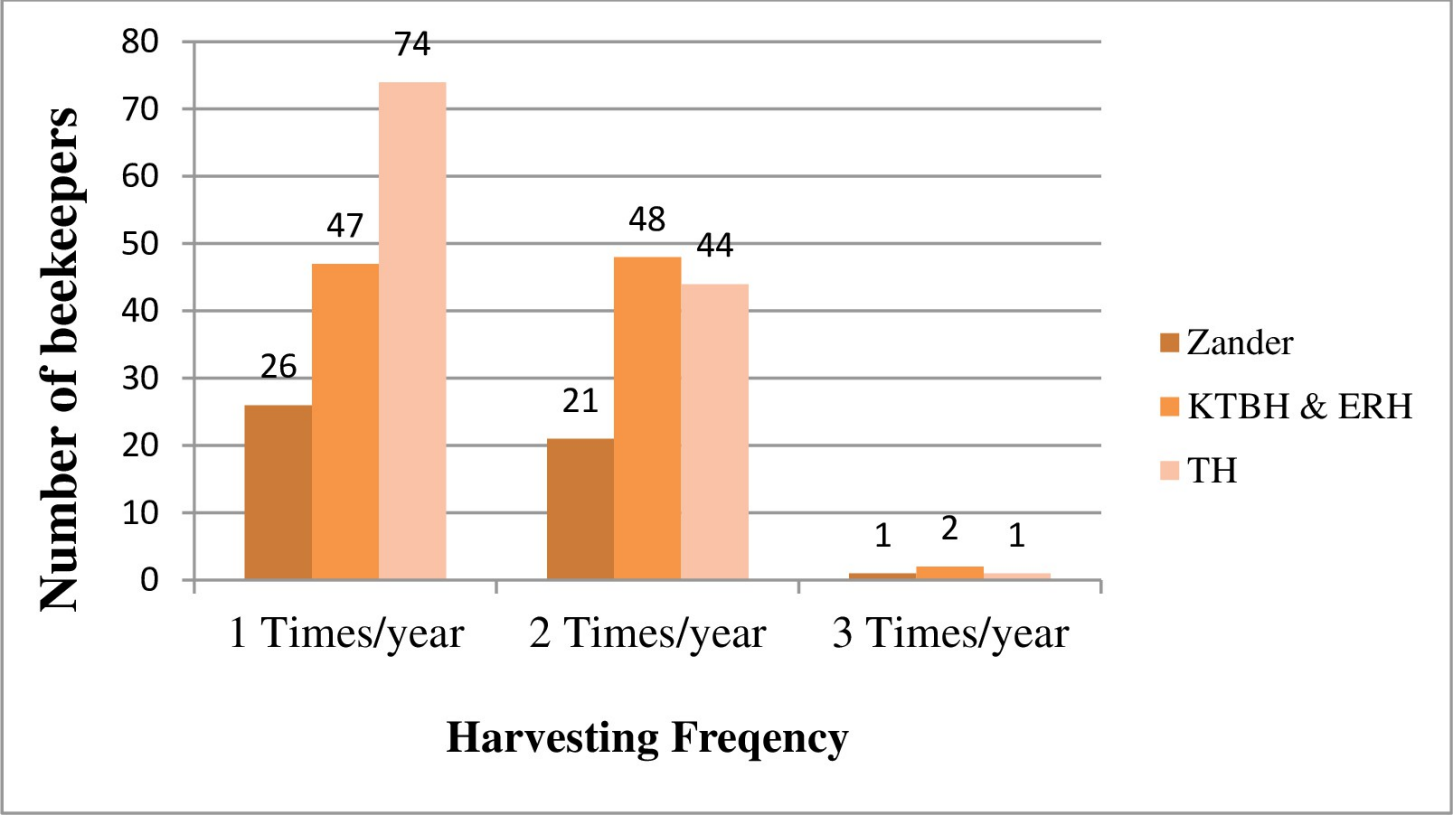
Honey harvesting frequency by hive type.

By following these floral calendars, the majority (53%) of the entire respondents’ harvested honey once a year from traditional and improved hives. However, about 44.4% of entire beekeepers harvested twice per year from different hives in Gedeo Zone. Exceptionally, 2.2% (4) beekeepers harvested three times per year from KTB and Zander hives in Bule and Wonago districts (Fig 2). The results further indicated that there was variability in honey harvesting frequency among the sampled districts. The majority in Wonago (60%) and Kochore (56.7%) districts (mid-altitude) were harvested once per year. Unlike to that, the majority (53.3%) of beekeepers in Bule district (highland) reported that they harvested two times in a year (Fig 2). Regarding hives types, most beekeepers experienced harvesting honey only one times a year from traditional, transitional (KTBH and Ethio-Ribrab hives), and frame hives (Zander) while comparatively less number of farmers practiced harvesting two time in a year from each types of hives (Fig 3).

## 4. Discussion

In the Gedeo zone, farmers have been practicing apiculture in the traditional, transitional (KTBH and Ethio-Ribrab hives or Mud hives) and frame hives (Zander) at a varied level (Fig 1). The present study revealed promising results in the beekeeping proportion. Honeybee colonies managed in the frame hive (12%) and transitional hives (25%) together account for 37 percent, while the traditional system were dominated by 63% in the sampled districts of Gedeo zone. This finding is different from the [15] reports of 96% for the traditional system, 2.8% for the frame hive, and 1.1% for the transitional hive at the national level.

Proper hive management and inspections were identified as crucial factors for honeybee health and productivity and are therefore considered as vital elements of successful beekeeping [27, 28]. These could be done better through improved beekeeping technologies. Regarding the adoption of improved hives, KTBH were largely disseminated to beekeepers and more occupied with colonized honeybees compared to Zander hives. Perhaps the KTBH was more preferred and better managed because it requires fewer skills to manipulate and does not need expensive equipment [29]. In general, beekeepers had low experience in improved hive management, particularly in frame hives (Zander). Because of poor colony management practices, nearly half of frame hives were colony-less compared to KTBH.

The honey yield in improved hives was very low compared with the national average [6], as well as the yields reported in other areas that possess similar or less beekeeping potential in the country [30–32]. This clearly proves that beekeepers lack practical skills in managing bees, particularly in improved hives, since apicultural resource were good enough to increase honey yield. The findings of the present study are different from the reports of [25] for all types of hives in the Gedeo zone sampled districts.

Among the identified honeybee floras, *Hygenia abyssinica*, *Bidens ghedoensis*, *Erythrinia abyssinica*, *Eucalyptus species*, *Cordia africana*, *Coffee arabica*, *Vernonia species*, *Susbania susban*, and *Persea americana* were major honeybee floras in the Gedeo zone. These bee plants were most frequently rated by over fifty percent of respondents in each district. The majority of bee plants in the Gedeo zone flower in three general periods: between February and March, May and July, and October and December. These resulted in three honey-harvesting seasons in the study area. February–March was the major active season in the Kochore and Wonango districts (mid altitude). However, October–December was the major honey flow season in Bule district (high land). Besides, May through July is a minor honey-harvesting period in both mid and high altitudes. According to [33], there are two common honey-harvesting seasons in Ethiopia: the first one starts from October to November, and the second one starts from April to June. In Southwest and Southeast parts of the country May–June is a major honey flow season [34]. The result of the present study was different from their findings for mid-altitude but inline for highland. The variability in the rainfall length and weather conditions between highland (Bule) and mid-altitude (Kochore and Wonago) is attributed to floral diversity and different flowering periods [20]. The Gedeo zone receives the longest and most heavy rainfall between July and October. Consequently, this period is not favorable for the foraging activities of worker bees and has a negative effect on nectar concentration. On the other hand, the area receives moderate rainfall between March and May, which fevered most flowering plants, particularly in the mid-altitudes of the sampled area.

Even if multiple floral calendars are known in the Gedeo zone, most beekeepers have been practicing honey harvesting once a year from improved and traditional hives. This is because of the wrong assumption of beekeepers that “jumping one season without harvesting should increase the honey yield per harvest”. In reality, they have been missing the honey crop that would otherwise be consumed by bees during the dearth season if not harvested. On the other hand, a significant number of farmers harvested two times in a year, with varied harvesting frequencies across different altitudes. Highland beekeepers have done well compared to midland beekeepers. However, this was not explained by the amount of honey yield in the improved hives, except for the traditional hives.

## 5. Conclusion

This study indicated that farmers in the Gedeo zone practice beekeeping in the traditional, transitional (KTBH and Ethio-Ribrab hives, or Mud hives), and frame hives (Zander hives). Traditional beekeeping is the dominant system in the study area. However, there is promising progress in the improved beekeeping (KTBH and Zander hives) regarding hive composition and beekeeping proportion. KTBH is largely disseminated and better managed by beekeepers compared to the Zander hive. Nevertheless, there was no significant variation in the honey yields between Zander hive and KTBH hives. The Gedeo zone had rich floral resources and a diverse floral calendar. February–March was the major honey-harvesting period, while May–July and October–December were the minor honey harvesting periods. However, most beekeepers have been practicing honey harvesting from improved hives once a year due to a lack of practical skills. Thus, honey yield in improved hives was low because of lower honey harvesting frequency and due to low experience in improved beekeeping. Therefore, we recommend that the local government should focus on educating beekeepers to enable them to utilize exhaustively the opportunities of the multi-floral season and improved hive technology to maximize honey yield in the area. Moreover, the majority of identified bee forages were tree species. Thus, further identification and documentation are required particularly for herbs and weeds, including pollen analysis.

## Supporting information

S1 Data (XLSX)

S1 Checklist (DOC)
