# Supplementary material for: Improved beekeeping practices, honey bee flora potential and flowering calendar in South Ethiopia
Source: PLoS One. 2024 May 29;19(5):e0304259. doi: 10.1371/journal.pone.0304259 (PMC11135692; doi:10.1371/journal.pone.0304259)
Supplement: S1 Checklist — (DOC) [file pone.0304259.s002.doc]

**DILLA UNIVERSITY, COLLEGE OF AGRIGULTURE AND NATURAL SCIENCES DEPARTMENT OF ANIMAL AND RANGE SCIENCE**

**Questionnaire**

**District…………………………………..Kebele…………….………….**

**A) BACKGROUND INFORMATION**

1) Respondent’s Names………………..…….. (2) Sex (a) Male (b) Female

3) Marital status (a) Married (b) single (c) Widowed

4) Age……….. (5) Education level and highest class attained………….……..

5) Land size_________ Annual Income___________ Credit access_______

6) Crop type cultivated ……………

8) Being member of cooperative_____________

9) Number times extension visit___________

**Part 1**

1. For how long you practiced beekeeping?
2. < 5 years B. 5-10 year C. 10-15 Years D. more than 15 years
3. Do you own improved bee hive?
4. Yes B. No
5. For what purpose do you keep honey bees?
6. To generate cash income B. To broaden food item C. For cultural and ritualistic ceremony
7. When do you start keeping bees in modern hives?
8. 2- 5 years ago B. 5-7 years ago C. 7-10 years ago D. > 10 years
9. From where do you gain beekeeping experience?
10. From parents B. Through training C. From neighbors’ D. From self- initiative

1. If you obtained experience through training, who provides training to you?
2. District Agricultural office B. NGOs C. Higher institutions D. Research institutions
3. Which type of hive do you own?
4. Zander B. langsroth C. other
5. How many hives do you own from each type and from where you get it?

| SN | Type of hives | No. of hive owned | Source(NGO, Go’s or purchased by beekeeper) | Colony- right | Colony-less | Honey yield /hive/year | How many times you harvest in a year |
| --- | --- | --- | --- | --- | --- | --- | --- |
| 1 | Zander |  |  |  |  |  |  |
| 2 | Langastroth |  |  |  |  |  |  |
| 3 | Other |  |  |  |  |  |  |

1. In which season of year you get more honey?
2. October to December B. May to July
3. How you provide box hive to your colony during colony transferring?
4. Brood box with foundation sheet fixed to all frame
5. Brood chamber and two suppers with few foundation sheet fixed to some frame
6. All box without foundation sheet fixed to all frame
7. Do you have modern beekeeping equipments?

| SN | Type BK equipments | Private | Used in common | Rent | Not available at all |
| --- | --- | --- | --- | --- | --- |
| 1 | Honey extractor |  |  |  |  |
| 2 | Wax molder |  |  |  |  |
| 3 | Chisel |  |  |  |  |
| 4 | Smoker |  |  |  |  |
| 5 | Queen excluder |  |  |  |  |
| 6 | Bee brush |  |  |  |  |
| 7 | Protective cloth |  |  |  |  |

1. How do you harvest honey from box (zander) hives?
2. By cutting combs with knife B. By removing frames and centrifuging with honey extractor
3. If you do not harvest honey from any of these type hive what is limitation in use of these technology?

| SN | Limitation in use of technology | high | medium | low | Rank |
| --- | --- | --- | --- | --- | --- |
| 1 | Lack of skill to use modern hive |  |  |  |  |
| 2 | Lack of modern BK equipments |  |  |  |  |
| 3 | Lack of bees wax |  |  |  |  |
| 4 | Inadequate support from Das |  |  |  |  |
| 5 | Due to absconding behavior of colony |  |  |  |  |
| 6 | Due to hive design problem |  |  |  |  |
| 7 | Absence of colony |  |  |  |  |

1. Do you have intermediate hives?
2. Kenyan top bar hive B. Tanzanian top bar hive C. Mud hive
3. How many hives do you own from each type?

| SN | Type of hives | No. of hive owned | Source(NGO, Go’s or purchased by beekeeper) | Colony- right | Colony-less | Honey yield /hive | How many times you harvest in a year |
| --- | --- | --- | --- | --- | --- | --- | --- |
| 1 | KTBH |  |  |  |  |  |  |
| 2 | TTBH |  |  |  |  |  |  |
| 3 | Mud hive |  |  |  |  |  |  |
| 4 | Traditional hive |  |  |  |  |  |  |

1. Which one of improved hive technology do you adopted very well?

| SN | Type of improved hive | Rate of adoption | | | |
| --- | --- | --- | --- | --- | --- |
|  |  | High | Medium | Low | Not at all |
| 1 | Zander hive |  |  |  |  |
| 2 | Kenyan top bar hive |  |  |  |  |

**Part 2**

1. What opportunities are available for beekeeping in your area?

| SN | Good opportunities for beekeeping | Rate of availability | | | Rank |
| --- | --- | --- | --- | --- | --- |
|  |  | High | Medium | Low |  |
| 1 | Surplus source of honey flora |  |  |  |  |
| 2 | Existence of highest colony population |  |  |  |  |
| 3 | Suitable agro ecological condition |  |  |  |  |
| 4 | Willingness of government to support beekeepers |  |  |  |  |
| 5 | Increasing Interest of investors and NGOs |  |  |  |  |

1. Is there application of insect cide or pest cide for the crop in your area?

A. Yes B. No

1. If your answer is yes, is there massive death in your colony at once?

A. Yes B. No

1. What are challenges you encountered in beekeeping work?

| SN | Major challenges in beekeeping | Level of constraints | | | Rank |
| --- | --- | --- | --- | --- | --- |
|  |  | High | Medium | Low |  |
| 1 | Unpleasant behavior of bees( absconding, swarming & aggressiveness ) |  |  |  |  |
| 2 | High price of improved BK technology |  |  |  |  |
| 3 | Deforestation and drought |  |  |  |  |
| 4 | Lack of skill |  |  |  |  |
| 5 | Lack of training institution |  |  |  |  |
| 6 | Indiscriminate application of agrochemicals |  |  |  |  |
| 7 | Honey bee disease, pest and predators |  |  |  |  |
| 8 | Poor extension service |  |  |  |  |
| 9 | Marketing constraint |  |  |  |  |
| 10 | Absence colony for starting beekeeping |  |  |  |  |

1. Which type of container do you use to store honey?
2. Ink container / Metal/
3. B. clay pot
4. Plastic jar
5. Glass jar
6. Plastic bag
7. What are major honey bee floras in your area? Please list them?
8. In which season you harvest honey from your honey bees?
